# Large bowel obstruction secondary to gallstones

**DOI:** 10.1093/jscr/rjab137

**Published:** 2021-05-18

**Authors:** Matelin Crosen, Paul Ghattas, Rovinder Sandhu

**Affiliations:** Department of Surgery, Lehigh Valley Health Network, Allentown, PA, USA; Department of Surgery, Lehigh Valley Health Network, Allentown, PA, USA; Department of Surgery, Lehigh Valley Health Network, Allentown, PA, USA

**Keywords:** gallstone ileus, bowel obstruction, cholecystocolonic fistula, cholelithiasis

## Abstract

Gallstone ileus is a rare complication of cholelithiasis, representing 1% of bowel obstructions. The usual site of obstruction is the ileocecal valve, though other sites have been reported. Here, we present two cases of gallstone ileus within the distal colon requiring surgical intervention. Two elderly females presented with vague abdominal symptoms secondary to large bowel obstruction from gallstone impaction. Both underwent attempted endoscopic retrieval without success. Patient 1 required laparoscopy converted to exploratory laparotomy with colotomy and removal of the stone. Patient 2 required partial colectomy and end colostomy formation due to acute sigmoid inflammation. Gallstone ileus is a rare cause of intestinal obstruction, though incidence increases with age. Cholecystocolonic fistulas allow stones to bypass the ileocecal valve, with the potential for impaction in the colon at the site of a stricture or tortuosity. Surgical intervention may be required in cases not amenable to successful endoscopic retrieval.

## INTRODUCTION

Gallstone ileus is a rare complication of cholelithiasis, with ~1–4% of small bowel obstructions found to be secondary to gallstone impaction within the gastrointestinal tract [1, 2]. Entry into the gastrointestinal tract most commonly occurs at the site of a cholecystoduodenal fistula (68%), with colonic (5%), jejunal, ileal and gastric fistulas also reported [3]. Obstruction occurs when the stone becomes impacted within the intestinal lumen; this typically occurs at the ileocecal valve (73%), though the site of obstruction may also be the jejunum (14%), colon (4–8%) or duodenum (5%) [3, 4]. Colonic obstruction from gallstone ileus tends to occur in the elderly female population [5]. Success with endoscopic therapy for colonic gallstone impaction is limited and surgical intervention is often required [3]. Below we present two cases of gallstone obstruction of the colon requiring surgical intervention for resolution of obstructive symptoms.

## CASE SERIES

### Case 1

**Figure 1:**
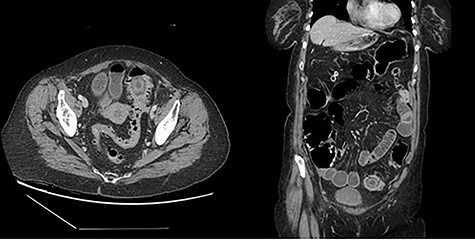
Representative cuts of Patient 1 CT scan.

The first patient is a 77-year-old female who presented to the emergency room with a 5-day history of nausea, vomiting, diarrhea and frequent belching. She had a past medical history significant for two episodes of diverticulitis treated non-operatively. She had never undergone abdominal surgery and she had no prior colonoscopy. She was afebrile and hemodynamically stable on evaluation. Her abdominal exam revealed mild distention without tenderness or peritoneal signs. No palpable masses were present on rectal exam. Her labs were remarkable for a mild leukocytosis of 12.4 thou/cmm. She underwent CT imaging of her abdomen and pelvis with oral contrast, which indicated the presence of a gallstone impacted in the proximal sigmoid colon with no contrast passing beyond this location; associated mild small bowel distention and pneumobilia suggested a cholecystoduodenal fistula (Fig. 1).

**Figure 2:**
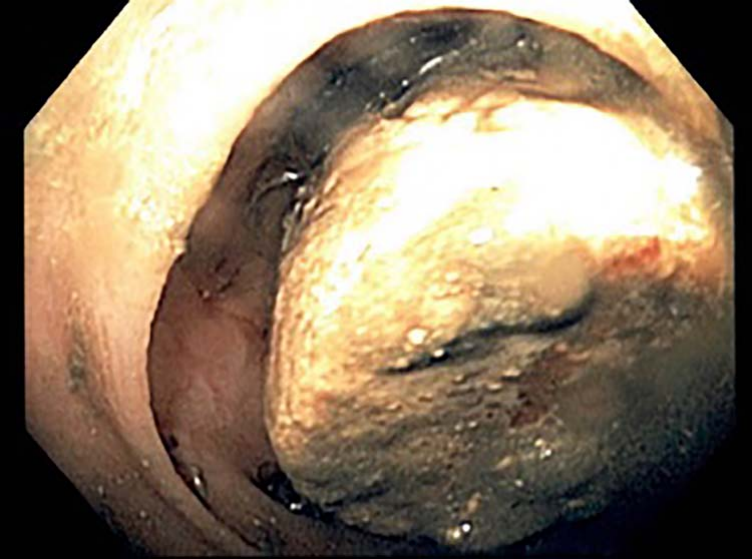
Image of gallstone from Patient 1 colonoscopy.

She was admitted and fluid rehydration was provided. Her symptoms improved and her abdominal exam remained benign. Daily abdominal radiographs were obtained to monitor transit of the gallstone; however, by hospital Day 3, she was unable to pass the stone and radiograph imaging failed to reveal movement beyond the location seen on admission CT. She was consequently taken to the endoscopy suite for flexible sigmoidoscopy. The large mobile gallstone was encountered at 40 cm (Fig. 2) and was grasped with a snare but was unable to be extracted beyond the rectosigmoid junction, which appeared narrowed. The procedure was then aborted.

**Figure 3:**
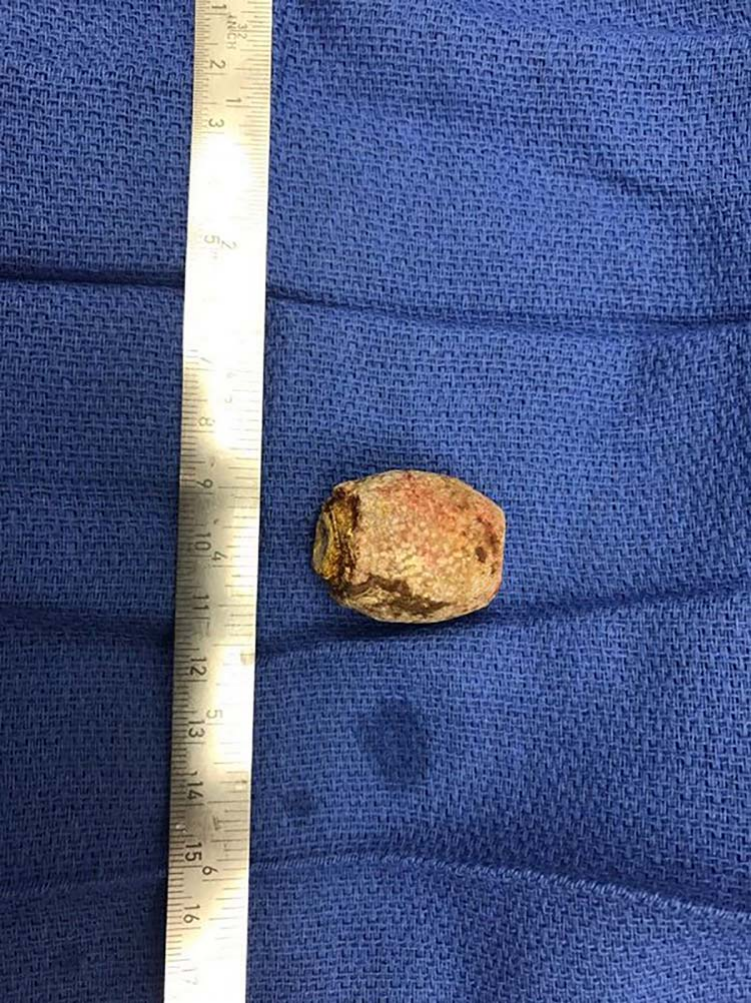
Patient 1 gallstone.

**Figure 4:**
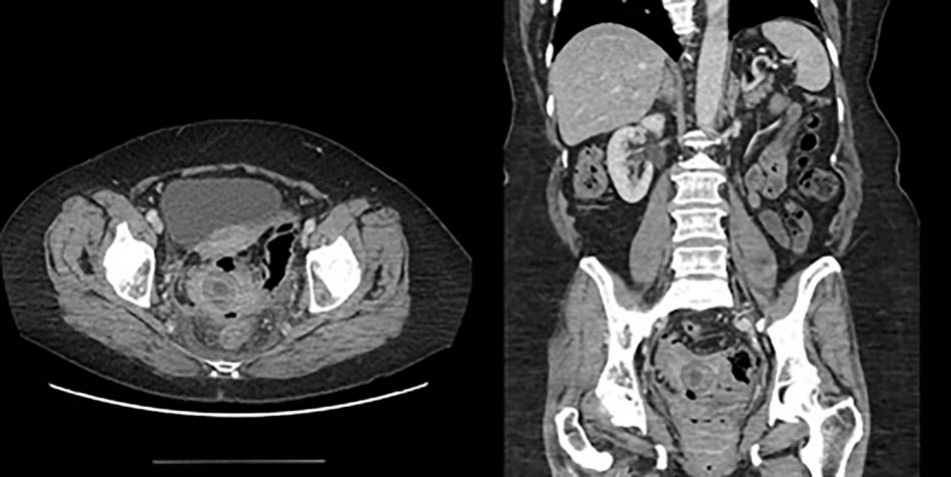
Patient 2 representative CT scan cuts.

The following day, sigmoidoscopy was repeated with attempts to grasp the stone with retrieval basket; although this was successful, the stone was unable to be brought through the narrowed rectosigmoid junction. Several attempts were made to fracture the stone to facilitate removal but were unsuccessful and the procedure was aborted. On hospital Day 5, the patient was taken to the operating room for a diagnostic laparoscopy. The stone was located in non-inflamed colon and attempts were made to laparoscopically manipulate the stone beyond the area of narrowing; colonoscopy with gentle insufflation was performed simultaneously without success. A small lower midline laparotomy incision was made and again the stone was unable to be pushed beyond the stricture. A segment of colon proximal to the stone was then extracorporealized and a small colotomy was made; the 28-mm gallstone was then milked in a retrograde fashion and was removed via the colotomy which was then repaired primarily (Fig. 3). The gallbladder was left *in situ*. The patient tolerated the procedure without difficulty. The patient’s post-operative course was uncomplicated. She recovered well and was discharged after return of bowel function.

### Case 2

The second patient is a 69-year-old female who presented to the emergency room with complaints of obstipation and abdominal pain. She reported a 2-month history of intermittent sudden onset abdominal pain accompanied by abrupt bowel movement and nausea. At the time of presentation, she reported constipation for 5 days; she had tried fiber and suppositories without relief. Her past medical history included hypertension and irritable bowel syndrome. She had no prior abdominal surgeries or colonoscopies. Upon evaluation, she was afebrile and hemodynamically stable. Her abdomen was softly distended with localized left lower quadrant tenderness and no peritoneal signs. Her rectal exam was unremarkable. She had a mild leukocytosis of 12.9 thou/cmm. Abdominopelvic CT with intravenous contrast was obtained and revealed inflammatory stranding consistent with acute diverticulitis with a lamellated intraluminal mass in the sigmoid colon in the region of inflammation as well as pneumobilia (Fig. 4); these findings led to the diagnosis of a gallstone impaction with resulting large bowel obstruction.

The patient was admitted, started on intravenous antibiotics and provided fluid resuscitation. Shortly after admission, she began passing flatus and the decision was made to observe her with serial abdominal exams in hopes that treating the acute diverticular inflammation would permit passage of the stone; however, by hospital Day 3, the stone remained partially obstructing and the patient required surgical intervention. Upon induction of anesthesia, flexible sigmoidoscopy was performed for diagnostic confirmation and possible removal of the gallstone. The stone was encountered at 40 cm but could not be retrieved or fragmented, so a laparotomy was performed. Her sigmoid colon was noted to be very redundant and tortuous, and the gallstone was palpable at the junction between the descending and sigmoid colon. There was a large degree of surrounding inflammation, consistent with the findings on CT. Due to the inability to pre-operatively prep the colon secondary to her bowel obstruction and the degree of inflammation that was present, a partial colectomy and an end colostomy was performed. The gallbladder was left *in situ*. The patient tolerated the procedure without difficulty.

Her post-operative course was without complication; she quickly gained ostomy function and tolerated a diet without difficulty. The pathologic evaluation of the colon specimen was consistent with diverticulitis and focal ulceration with a 36-mm gallstone present. Approximately 3 months after her index case, the patient underwent colonoscopy via her stoma in preparation for reversal of her colostomy. An area was noted in the transverse colon that was thought to represent the site of healed cholecystocolonic fistula.

## DISCUSSION

Both patients had diverticular pathology which likely contributed to the impaction of the gallstone within the colonic lumen. Patient #1 had two documented cases of uncomplicated diverticulitis which were successfully treated in a non-operative fashion. The presence of narrowing at the rectosigmoid junction, which prohibited the endoscopic removal of the gallstone, is believed to represent a mild structuring from the diverticular episodes. Patient #2 had a large degree of inflammation consistent with acute diverticulitis on both imaging and operative evaluation; presumably, the presence of the gallstone within the area may have contributed to the degree of inflammation present. Patient #2 did not have a documented history of diverticulitis; however, she did have a diagnosis of irritable bowel syndrome which upon review of the chart had been made without imaging or colonoscopy. Consequently, her chronic intermittent low-grade abdominal pain may have been episodes of subclinical diverticulitis. Additionally, her tortuous sigmoid colon was likely a factor in the difficult transit of a large cholelith.

No consensus exists in the literature as to the ideal management of colonic gallstone ileus. Systematic review by Farkas *et al.* [4] encompassing 38 patients revealed attempts at endoscopic or conservative management in 61% of patients, with 26% of the patients resolving with these measures alone; 74% of the patients required operative intervention, though the review is limited by a small (38 total) patient sample. Endoscopic management of gallstones includes both retrieval and reports of successful lithotripsy [4, 6]. Surgical options include manual/laparoscopic antegrade evacuation of stone, proximal enterolithotomy, bowel resection with or without ostomy creation, or proximal diversion with loop ostomy creation [4]. Surgical options should be guided by patient factors including age and comorbidities, as well as the presence of perforation, intraabdominal contamination or bowel inflammation at the time of operative intervention.

## CONCLUSION

Colonic obstruction from gallstone ileus is a rare complication of cholelithiasis, typically presenting in elderly female patients and classically secondary to a cholecystocolonic fistula. In the stable patient, attempts at endoscopic management may be possible as evidenced by the literature [4]; when endoscopic management fails, surgical intervention is warranted to relieve the obstruction. Here, we presented two cases of sigmoid colon obstruction from gallstone ileus. One patient had an isolated obstruction from a diverticular stricture and was successfully managed with proximal colotomy and stone removal. The second patient presented a more challenging scenario due to marked inflammation of the colon from concurrent acute diverticulitis and required Hartmann’s procedure for resolution of symptoms. Both patients recovered uneventfully.

## CONFLICT OF INTEREST STATEMENT

No conflicts of interest to disclose. No previous presentations or submissions.

## FUNDING

No funding to disclose.
